# Smarter faster just-in-time hemorrhage control: A pilot evaluation of remotely piloted aircraft system delivered STOP-THE-BLEED equipment with just-in-time remote telementored deployment

**DOI:** 10.1016/j.heliyon.2023.e12985

**Published:** 2023-01-18

**Authors:** Andrew W. Kirkpatrick, Jessica L. McKee, John M. Conly, Kristin Flemons, Wade Hawkins

**Affiliations:** a Tele-Mentored Ultrasound Supported Medical Interventions (TMUSMI) Research Group, Calgary, Alberta, Canada; b Department of Critical Care Medicine, Canada; c Department of Surgery, Canada; d Trauma Program, Foothills Medical Centre, Calgary, Alberta, Canada; e University of Calgary, Canada; g Department of Medicine, University of Calgary, Calgary, Alberta, Canada; h W21C, O’ Brien Institute for Public Health University of Calgary, Calgary, Alberta, Canada; i Centre for Innovation and Research in Unmanned Systems (CIRUS), Southern Alberta Institute of Technology (SAIT), Calgary, Alberta, Canada

**Keywords:** Resuscitation, Hemorrhage control, Remotely piloted aircraft systems, Remote telementoring, Prehospital

## Abstract

**Introduction:**

Remotely Piloted Aircraft Systems (RPAS) can access patients inaccessible to traditional rescue. Just-in-time remote telementoring (RTM) of naïve users to self-care could potentially address challenges in salvaging exsanguination in remote environments.

**Methods:**

An exsanguination self-application task was established in a wilderness location. Three volunteers-initiated distress calls to prompt RPAS precision delivered STOP-THE-BLEED kits, after which a remote mentor directed the volunteers how to self-care.

**Results:**

Limited connectivity prevented video, however each volunteer delivered images and initiated conversation with the mentor pre-RPAS arrival. Thereafter, all subjects were able to unpack and deploy hemorrhage control adjuncts under verbal direction, and to simulate self-application. All subjects were able to successfully apply wound-clamps, tourniquets, and pack wounds although one had insufficient pressure.

**Discussion:**

RPASs can deliver supplies long before human rescuers, and communication connectivity might allow remote mentoring in device application. Further development of technology and self-care paradigms for exsanguination are encouraged.

## Highlights

• Bleeding to death is the most preventable cause of post-injury bleeding.
• The earliest possible hemorrhage control is required to save lives, especially with self-care.
• Potentially self-applied hemorrhage control adjuncts can be delivered by RPAS before traditional human responses.
• Conscious victims may be able to utilize unfamiliar hemorrhage control adjuncts with remote telementored direction.
• Further research is urgently required into the remotely piloted aircraft systems delivery of hemorrhage control adjuncts.

## 1 Introduction

The STOP THE BLEED program is a remarkable initiative led by the American College of Surgeons and the Office of Homeland Security that recognizes both common sense and individual responsibility in an attempt to ensure that no one ever bleeds to death unnecessarily [[1], [2], [3]]. This is a realistic concern in modern society however, as exsanguination is the most frequent and potentially preventable cause of death after traumatic injury and thus the highest priorities in prehospital care must be in providing early and effective hemorrhage control [[4], [5], [6], [7], [8], [9], [10]]. Recent paradigms such as the STOP THE BLEED campaign recognize increased responsibility for the earliest hemorrhage-control application whether provided by first responding law-enforcement, lay personal, or even the victim themselves [1,[11], [12], [13], [14], [15]]. The TeleMentored Ultrasound Supported Medical Interventions (TMUSMI) Research Group has a mandate to focus on this latter paradigm; empowering the victim of trauma or critical illness to assist when possible, in their own resuscitation through simple off-the-shelf telecommunications technologies that are increasingly present in most households or pockets [[15], [16], [17]].

A logistical limitation in expecting anybody to be able to assist in their own life saving hemorrhage control is however the availability of standard resuscitative adjuncts. These mechanical adjuncts to arrest hemorrhage, especially extremity hemorrhage, are not complex, but are not typically carried by most citizens in their daily activities. However, when someone becomes an unexpected victim, simple adjuncts such as wound packing, tourniquets, wound clamps, and if needed a smart phone-based communications platform may be lifesaving. Remotely Piloted Aircraft Systems (RPAS) have revolutionized modern conflict allowing the Ukraine to persevere in a “David and Goliath” situation [18]. RPASs might also fundamentally change prehospital care in delivering life-saving medical equipment before any prehospital provider formally trained to use such equipment arrives. Drone delivery would be followed with Telemedical guidance to the victim to use the delivered equipment upon themselves rather than simply waiting and dying.

## 2 Materials and methods

The study was conducted after informed consent was obtained from all participants as part of our ongoing studies into the Marriage of Hyper-Realistic Surgical Training for First responders with Tele-presence Mentoring (REB14-0634), approved by the Conjoint Health Research Ethics Board of the University of Calgary.

**Fig. 1:**
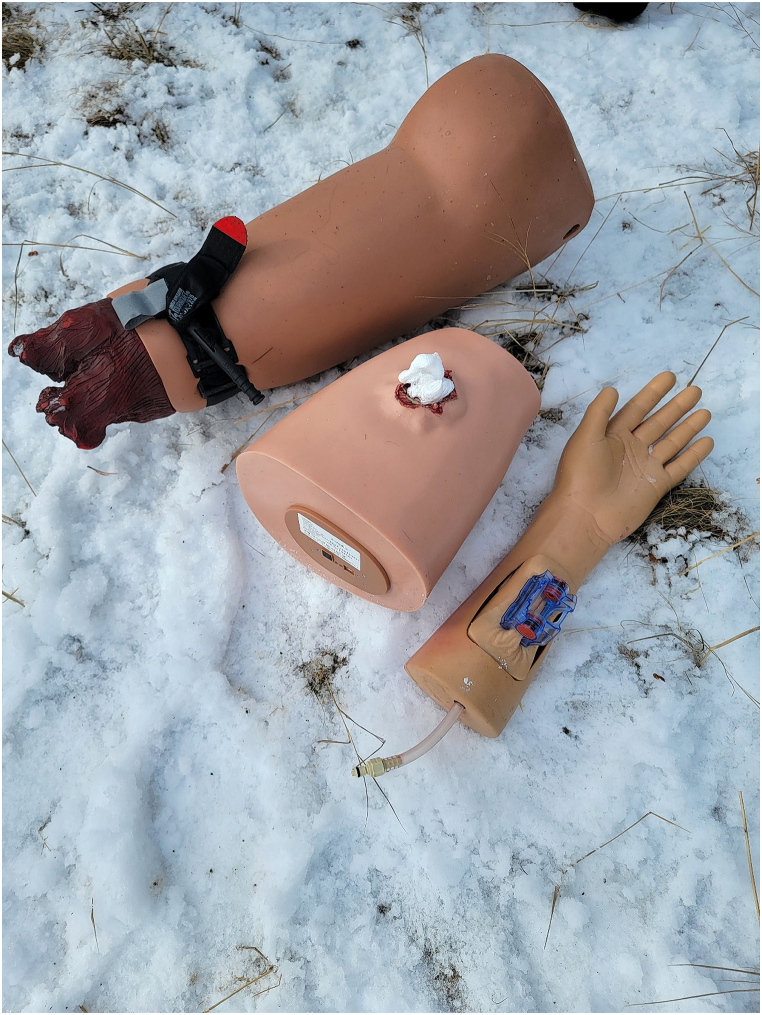
Exsanguination simulators used in the self preservation task.

**Fig. 2:**
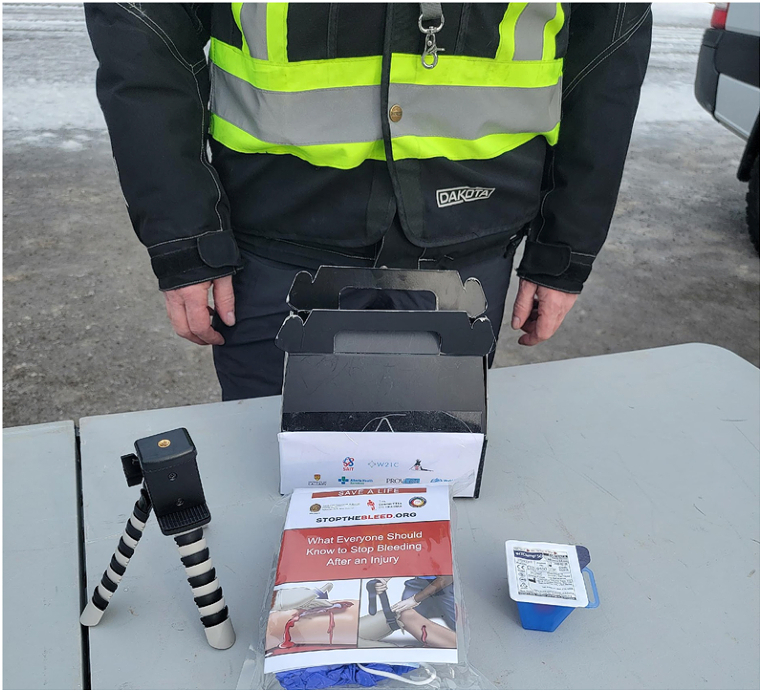
Augmented Self Preservation Stop-the-bleed kit being prepared for “drone-delivery”.

**Fig. 3:**
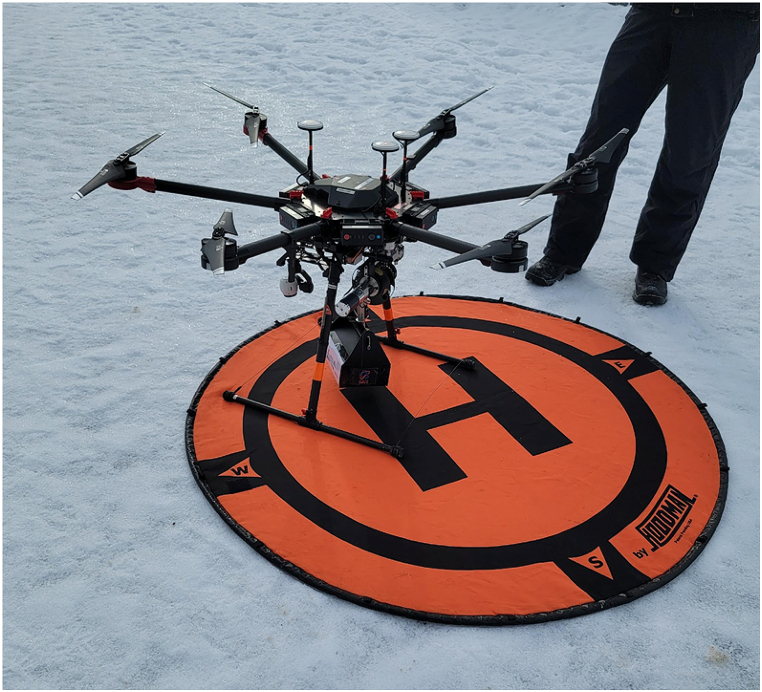
DJI Matrice 600 Pro Uncrewed Aerial Vehicle carrying Augmented Self Preservation Stop-the-bleed kit.

A simulated self-hemorrhage control task was created by transporting 3 extremity exsanguination simulators to a remote location in Kananaskis Country, Alberta, Canada, 94 km distant from our tertiary referral centre in winter conditions. The simulators were a; i) Hapmed tourniquet trainer, (Chisystems, Plymouth Meeting PA), ii) Z-Medica Hemorrhage Control Packing Trainer with Biofeedback (Z-Medica,Wallingford, CT), and iii) the Sawbones extremity exsanguination simulator (1534 Arm Trainer, Pacific Research Laboratories Inc, Vashon Island, Washington) (Fig. 1.). For completion of the self preservation task, the Hapmed required application of a standard tourniquet (Combat Application Tourniquet (CAT) Tourniquet, North American Rescue Co, Greer, SC), the Z-Medica the insertion of wound packing with Quick clot gauze (Quickclot, Littleton, NH), and the Sawbones the application on a wound clamp (ITClamp, Innovative Trauma Care Corp, Edmonton, Canada). Hemorrhage Control adjuncts were prepared for a RPAS delivery by purchasing on “off-the-shelf” American College of Surgeons Personal Stop the Bleed Kit (PSTBK) (Personal STOP THE BLEED® Kit | STOP THE BLEED® - American College of Surgeons (bleedingkits.org) which contained one STOP THE BLEED® Instructional booklet, one CAT tourniquet, QuikClot® Z-Fold Dressing, 4 in. Responder Flat Dressing, one permanent marker, surgical gloves and a mask. This kit was supplemented with an ITClamp, and a phone stand (Ubeeze Tripod Stand, City of Industry, Ca)) all packed in a drone payload box (Fig. 2.). The RPAS utilized was the DJI Matrice 600 Pro (SZ DJI Technologies Corp, Shenzhen, Guangdong, China), with a flight speed of 10 m per second (36 kph or 22 mph) (Fig. 3.), flown and under the control at all times of the Centre for Innovation and Research in Unmanned Systems (CIRUS), from the Southern Alberta Institute of Technology (SAIT), Calgary, Alberta. Each participant was required to use their own phone during the drill to call the telementor, with the assumption that most victims dying of trauma in the contemporary world have a smart phone on their person [15]. Although instructions were included within the PTSBK they were not read by the participants although they provide a potential resource for disconnected situations.

Volunteers simulated the role of an injured isolated victim and initiated a cell phone call to the mentor in Calgary. The victim's location was communicated with the drone team and the coordinates were entered into the drone. Due to limited cell phone coverage, video-communication with the mentor was not possible, but the “victims” were able to describe their simulated injuries and to send digital images of the injuries to the remote mentor. The remote mentor initiated RPAS-delivery of the PSTBK, which was in the area of the victim, but not in their line of site. Thereafter the RPAS flew to the “victims” geographic location, hovering over the victim using a downward facing camera and lowered the PSTBK within arms reach of the victim. Following successful delivery, the “victim” opened the payload container, and continued their conversation with the remote mentor, who guided the “victims” to unpack the kit and to utilize the hemorrhage control supplies on each of the simulators. After the scenario, participants completed a formal post-test survey and were debriefed.

## 3 Results

**Fig. 4:**
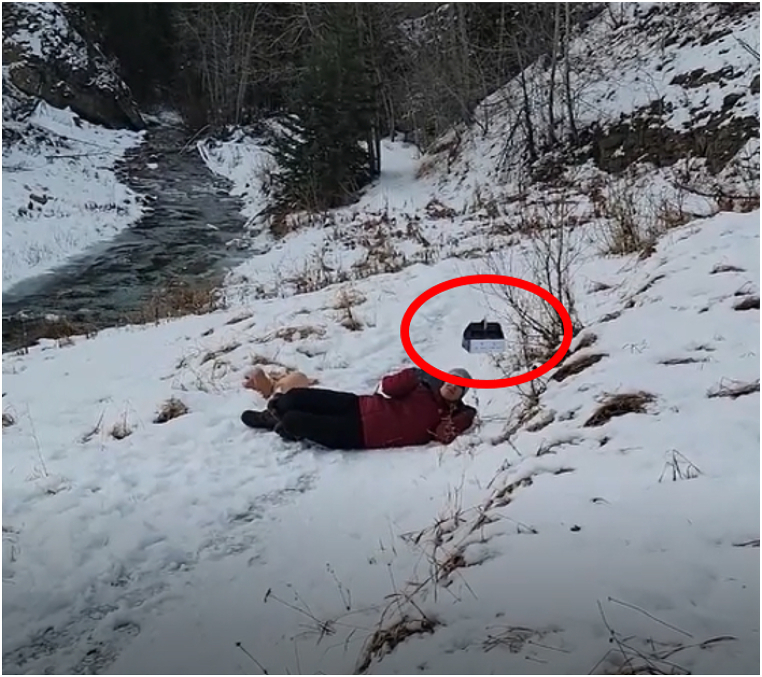
Drone delivers the Personalized Stop the Bleed Kit (PSTBK) within arms length of the victim.

**Fig. 5:**
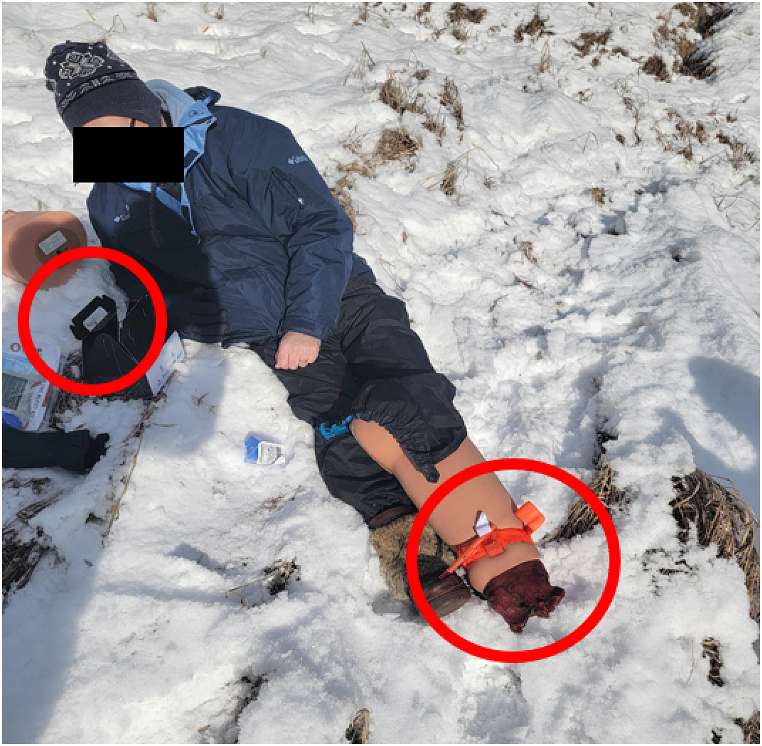
Victim after applying the tourniquet to simulated own limb while communicating with remote mentor on their own smart phone.

Three volunteers participated, all of whom had no practical familiarity with the hemorrhage control devices. All participants were able to initiate direct cellular voice communication with the remote mentor, although none were able to establish video communication (Table 1). The drone pilot was able to deliver the PSTBK within arms reach of all “victims” (Fig. 4) (**Supplemental video file**). Thereafter, all volunteers were able to unpack the kit and to deploy the hemorrhage control adjuncts under the verbal direction of the mentor. Each volunteer was instructed to put their cell phone on speaker mode and attach the cell phone holder that was provided in the kit allowing them to be hands free. The first instruction addressed by all was tourniquet application. Each volunteer applied the tourniquet roughly 4 finger breaths above the wound. The biggest challenge with tourniquet application was ensuring that the tourniquet was not only applied at the right location but was also tight enough (Fig. 5). Thereafter each participant was instructed to remove the QuikClot® gauze from the package and to pack the wound using a finger-over-finger technique. The biggest challenge was proper packing technique and the subsequent holding of pressure for 3–5 min. Finally, the mentor instructed each volunteer to remove the iTClamp from the package and thereafter apply it to the Sawbones simulator in order to address the open wound on the arm laceration, without any iatrogenic injury from the skin contact needles inherent to the ITClamp design.

**Table 1:** Subjective participant evaluation of remote telementoring.

Study Parameter	Subjective Median Opinion^1^
**Connectivity**	Cellular Network Provider
Subject 1	Rogers
Subject 2	Telus
Subject 3	Koodo (Telus)
Able to support video communication	0/3
Able to support asynchronous image transmission	3/3
Able to support real term audio communication	3/3
**Human Factors**	
Could hear the mentor clearly	4 (agree)
Mentor made the task easier	5 (strongly agree)
Was comfortable without mentor	2 (disagree)
Stop-the-bleed Concept has merit	5 (strongly agree)
Participant would have preferred a video demonstration	2 (disagree)

**Legend:** °Opinions were based on a 5-point Likert Scale ranging from 1 (strongly disagree); 2 (disagree); 3(Neutral), 4 (agree), to 5 (strongly agree).

Supplementary video related to this article can be found at https://doi.org/10.1016/j.heliyon.2023.e12985

The following is the supplementary data related to this article:

Video 1 1 Video 1

With direct communication from the remote subject matter expert mentor, all procedures were performed successfully by the “victim” while in a seated position and verified by an on-site study coordinator. All (100%) of the tourniquets and ITClamp were applied successfully and there were no needle pokes or safety issues with the ITClamp. While all 3 participants were able to pack the wound successfully, one of the participants did not use consistent pressure to pack the wound. Each of the volunteers provided feedback after the scenario was completed (Table 1). The volunteers agreed that they could hear the mentor clearly, strongly agreed that the mentor made the task easier and that they would not have been comfortable performing the tasks without the mentor. All saw the Stop-the-bleed program as having merit and preferred the live mentor over the idea of watch an instructional video.

## 4 Discussion

Among many take-home messages, the most critical concept validated was the ability for a drone to deliver life-saving adjunctive hemorrhage control supplies to a simulated victim in a remote location much sooner than standard human rescue responses could ever be expected to physically respond. A further critical aspect of the expedited drone response was the utilization of the “victims” own personal smartphone to empower the “victims” own self-preservation efforts remotely supported by a remote on-line trauma/critical care surgeon. Although not utilized in this simulation, smart phones may also expedite discovery of victims through their geolocating capabilities. Exsanguination is critically time dependant and the best care applied too late is irrelevant to a dead patient. Duchesne has emphasized that the only quantity more valuable than blood is time, as once lost time is irreplaceable [19].

A realistic appraisal is that trauma care and most importantly trauma outcomes are dramatically influenced by a tyranny of distance where those residing outside of established population centres have worse outcomes [20,21]. Drones may dramatically reduce this tyranny of distance by removing the need for a physical human response [22] and may even be pre-positioned in a standby capacity in a fashion that wound be completely impractical for human first responders. While drones have been previously described as having the ability to deliver medications, blood products, and even human organs for transplant [22,23], we believe our group is the first to describe drone delivered interventional capabilities, other than drone delivered defibrillation [24,25].

CIRUS has previously done substantial work considering how to deliver critical medical supplies to injured but conscious victims. This has involved testing remote landing, tethered and parachute delivery approaches. In situations where the RPAS was either too far from the operator or suffered from urban canopy/topographic variation, radio signal degradation between the RPAS operator and the RPAS may prevent safe remote landing. Parachute delivery was also deemed to be unsuitable due to the limited accuracy of the payload drop and the ‘victim’ being unable to move to the location of the payload drop. Thus, the winch system was selected since it allowed the pilot in command to maintain radio connection with the drone and drop the payload very accurately, often within a foot of the victim (Fig. 4.).

We perceive that greatest logistical limitation to RPAS delivered interventional equipment being helpful, is the fact that inexperienced prehospital providers and especially injured laypersons would not be expected to be able to safely use such equipment. However, the TMUSMI group has a long experience in remotely guiding inexperienced non-physician to perform interventional tasks guided by a distant mentor, and we have been continuously surprised by the great potential of motivated persons to follow directions to perform simulated but advanced medical tasks [26,27]. Objective post simulation evaluations have further emphasized the fact that an engaged and empathetic remote mentor can reduce stress and make the inexperienced provider more comfortable when operating beyond their comfort zone [26,28], although conversely a poor mentor experience may be counterproductive [29,30].

A novel characteristic of this paradigm however involves supporting potentially dying victims to save themselves. Interventional self-care is a relatively poorly discussed concept in the medical literature but is intuitive throughout human history. However, most humans have strong innate survival instincts and have been noted to have performed remarkable interventions when there were no alternatives to save themselves [31,32]. The TMUSMI group postulates that if an exsanguinating victim was able to communicate with a remote hemorrhage control expert after drone delivery of the hemostatic adjuncts, then the remote expert could direct a cooperative and motivated victim to potentially save themselves. This paradigm would require at minimum the victim retains consciousness although whether complete or partial limb mobility is required remains a pertinent unknown. Even if victims have had exposure or teaching involving hemorrhage control techniques, a remote mentor is presumably invaluable as studies have shown poor retention of hemorrhage control skills by non-physicians. When a group of potential lay responders who had previously undergone the American College of Surgeons Bleeding Control Course were reassessed, adequate hemorrhage control performance was delivered less than 4% of the time [33]. Further, poor retention of hemorrhage control skills as soon as three months after training was also noted by both Jafri and Goralnick in controlled studies [34,35].

It is remarkable and sobering that only 94 km from a major urban population centre, sufficient internet connectivity to support real-time video communications was impossible as no participant could obtain this on their smart phones. Despite this, all could engage in real-time audio conversation with the mentor and were able to send digital images of the simulated injuries that aided the remote expert in appreciating that the simulated injuries of the “victim” and the hemostatic adjuncts that were drone delivered. It is relevant that all participants felt that the remote mentor “made the task easier” and agreed that the Stop-the-Bleed campaign has merit, and that they specifically disagreed that they would have been comfortable without the mentor being available (Table 1).

The TMUSMI group specifically notes the comments of the participants that they disagreed with the suggestion that a video demonstration of the tasks to be performed would have been helpful or better (Table 1). This thread specifically refers to the concept of video modelling (VM), which is defined a form a behavior modelling that involves the demonstration of desired behaviors, outcomes, and attitudes through active visual representations [36]. Such a technique will be required for disconnected and austere environments without phone or internet connectivity, where the guidance must be autonomous. Randomized trials by the TMUSMI group have suggested that VM has remarkable potential to support less experienced prehospital providers needing to perform heroic life-saving interventions without any other support, although both remote telementoring and VM have attributes and the ideal prehospital support system for interventions in catastrophic situations may be a hybrid approach. However, in many austere and disconnected environments or for trauma care beyond Low Earths orbit, VM may be the only option available and thus this paradigm should be more stringently studied for use both on and beyond the planet earth [36,37].

The limitations of this study are the small number of participants involved reflecting the reality of personnel being available to travel to the drone testing site given the extremely unpredictable nature of the administrative, cultural, and bureaucratic oversight on the project. The investigators would like to have involved many more participants in the evaluation but take solace from the fact that all three volunteers had contiguous opinions regarding the ease and potential utility of the paradigm and technology. This initial experience also includes a distinct conservative bias with the internet connectivity being very limited even within a 100 km radius of a major metropolitan Canadian city. As satellite technology increasingly delivers enhanced broad-band internet to the entire planet so there is great potential for the ease of usability to continually improve including remote and mountainous areas. Further RPAS may also in the future bring their own connectivity capabilities with them to remote sites. This would allow global scope and the ability of expert mentors anywhere in the world to support local RPAS delivery capabilities in austere and less developed areas of Earth.

## 5 Conclusions

A remotely piloted aircraft delivery device (RPADD) is able to quickly deliver life-saving medical supplies to dying victims with short windows of survivability. Self-preservation and hemorrhage control may often require self-interventions which may be remotely guided by a distant expert. Further research into the logistics and human factors of such interventions are required to ensure that the distant mentor is both empathetic and successful.

## Author contribution statement

**Andrew W Kirkpatrick**: 1- Conceived and designed the experiments, 2 - performed the experiments, 3 - analyzed and interpreted the data, 4 - contributed materials, and 5 - wrote the paper

**Jessica L McKee**: 1 - Conceived and designed the experiments, 2 - performed the experiments, 3 - analyzed and interpreted the data, 4 - contributed materials, and 5 - wrote the paper

**John M Conly**: 2 - Performed the experiments, 3 - analyzed and interpreted the data, and 5 - wrote the paper

**Kristin Flemons**: 2 - Performed the experiments, and 5 - wrote the paper

**Wade Hawkins**: 2 - Performed the experiments, 4 - contributed materials, and 5 - wrote the paper

## Declaration of competing interest

This work was partially supported by the Andrew W Kirkpatrick Professional Corporation.

JL McKee has consulted for Innovative Trauma Care, SAM Medical, Aceso, Acelity (3 M/KCI), ZOLL Medical and the Andrew W. Kirkpatrick Professional Corp. She has previously disclosed a personal relationship with AW Kirkpatrick.

AW Kirkpatrick has consulted for the Zoll, Acelity (3 M/KCI), CSL Behring, Innovative Trauma Care and SAM Medical Corporations, and the Statesman Group of Companies, and is the PI of a randomized trial partially supported by the Acelity Corporation and has previously disclosed a personal relationship with JL McKee.

No other author reported any Disclosures.
